# The association between adherence to alternative healthy Diet Index (AHEI) and severity, disability, duration, and frequency of migraine headache among women: a cross-sectional study

**DOI:** 10.1186/s12937-023-00867-4

**Published:** 2023-08-23

**Authors:** Pardis Khalili, Atieh Mirzababaei, Faezeh Abaj, Shakila Ansari, Asma Rajabi Harsini, Mahya Mehri Hajmir, Cain C. T. Clark, Khadijeh Mirzaei

**Affiliations:** 1 Department of Nutrition, Science and Research Branch, Islamic Azad University, Tehran, Iran; 2 Department of Community Nutrition, School of Nutritional Sciences and Dietetics, Tehran University of Medical Sciences (TUMS), Tehran, Iran; 3 -Nutrition and Food Security Research Center, Department of Community Nutrition, School of Nutrition and Food Science, Isfahan University of Medical Sciences, Isfahan, Iran; 4 Department of Clinical Nutrition, School of Nutritional Sciences and Dietetics, Tehran University of Medical Sciences, Tehran, Iran; 5 Centre for Intelligent Healthcare, Coventry University, Coventry, CV1 5FB UK

**Keywords:** Alternative healthy Diet Index, Severity of migraine, Duration of migraine, Frequency of migraine

## Abstract

**Background:**

Migraine is a common brain disorder characterized by recurrent seizures lasting between 4 and 72 h. Dietary factors can affect migraine headaches. The Alternative Healthy Diet Index (AHEI) is a measure of diet quality and adherence to healthy dietary patterns. This study aimed to assess the association of adherence to AHEI and severity, disability, duration, and frequency of migraine headaches.

**Methods:**

In this cross-sectional study, 266 women who suffered from migraines were selected. Usual dietary intakes were assessed using a semi-quantitative, 147-item, food frequency questionnaire (FFQ). AHEI was calculated based on FFQ. The Migraine Disability Assessments (MIDAS) and Visual Analog Scale (VAS) questionnaires were used to evaluate migraine disability, severity and the pain, duration and frequency of headaches.

**Results:**

People with high adherence to AHEI, compared with low adherence, had a 43% reduction in headache duration in the crude model (OR = 0.57; 95% CI 0.34, 0.97; P = 0.03), which remained significant after adjusting for potential confounders (OR = 0.56; 95% CI 0.31, 0.99; P = 0.04). No association was found between the frequency of migraine and AHEI in both crude and adjusted models (OR = 1.19; 95% CI 0.66, 2.14; P = 0.55). In addition, no significant association was found between high adherence of AHEI and odds of severe and moderate headaches (P > 0.05).

**Conclusion:**

The results of our study showed that people with high adherence of AHEI had a 43% reduction in duration of migraine. More studies are needed to evaluate and better understand this relationship.

## Introduction

Migraine is a common brain disorder characterized by recurrent seizures lasting between 4 and 72 h. Its pain characteristics can be attributed to moderate to severe unilateral throbbing pain, that worsen in physical activity, and can be concomitantly associated with nausea [1]. Approximately 12% of the world’s population has migraines [2], it is the third most common disease in adults worldwide, and the third leading cause of disability in people under 50y with a prevalence of 11.6% [3, 4]. Moreover, its prevalence in women (More than 20%) is twofold more than men (more than 10%) [5]. Migraine headaches are caused by various factors, such as diet, hunger, time of day, hormonal changes, family history, depression, stress, and irregular sleep patterns [6].

There are several factors involved in the pathogenesis of migraine, but the underlying cause is still unknown. Some studies have suggested the role of nerve damage in the brain, as well as the density and pressure of the nervous system, in causing migraines. Also, the role of food in engendering migraine should not be ignored, indeed, food may elicit pain through the effect of vasoconstriction and expansion on the nervous system [6]. Pickled fermented products, salty, monosodium glutamate in fast food, caffeine in coffee and chocolate, nitrate in food containing protein, aspartame, seafood, dairy, and foods containing tyramine, including red wine and alcohol, caffeine, smoked fish, old cheese, beans, onions, fruits such as figs, citrus fruits, avocados, and bananas are among the highest migraine-stimulating foods. It has also been shown that alpha-lipoic acid and eicosapentaenoic acid may play a role in reducing the severity and frequency of migraines [7]. The other factors include vitamin D deficiency, production of inflammatory agents and prostaglandins, production of serotonin from platelets, stimulation of norepinephrine production, and increased sensitivity to nitric oxide and hyperhomocysteine [8, 9]. People with migraines are more likely to develop cardiovascular disease and death [10], and therefore, control and management of this disorder is very important [11]. Drug regimen are used extensively, however, have major side effects [12], and so, identifying alternative treatment approaches is important. Some studies have shown that a variety of diets, including high-folate, low-fat, omega-3 and low-omega-6 fats, ketogenic, atkins, and low-sodium diets, may be a promising approach in managing migraines and reducing the severity of seizures [13]. A nutritious diet is an important factor that is relatively easily modifiable. Indeed, it has been reported that consuming more vegetables, fruits, and legumes is associated with a reduction in headache duration and intensity [14]. Additionally, nutrients are usually not consumed alone, where food groups consisting of several foods are consumed, which is assumed to be a factor for the existence of synergistic effects of complex food combinations [6]. The AHEI is a measure of diet quality and adherence to healthy dietary patterns, suggesting better recommendations for healthy diets to improve health observation to factors, as well as greater predictive power for chronic diseases. AHEI includes healthy foods, such as fruits, vegetables, whole grains, nuts and legumes, n-3 chain fatty acids, polyunsaturated fatty acids, fruit juice and sugar-sweetened beverages, red and processed meat, trans fatty acids, sodium, and alcohol [15]. Previous studies have shown that migraine is associated with inflammation and oxidative stress [16], and since high fiber, whole grains, vegetables, fruits, magnesium, which are known to improve systemic inflammation, as well as suppress the inflammatory process, and these foods are components of AHEI.

Although there have been studies on the effect of diet on migraines, to our knowledge, no study has investigated the impact of AHEI on migraines. In the present study, we sought to investigate the association between adherence to Alternative Healthy Diet Index (AHEI) and severity, duration, and frequency of migraine headaches among women.

## Materials and methods

### Study population

In this cross-sectional study, 266 women (18 to 50 years old) who suffered with migraines, who visited the brain and neurology clinics of Khatam al-Anbia and Sina hospitals and the professional headache clinic in Tehran, were selected The sample size was computed according to the following formula: n = [(Z1 − α + Z 1 − β) × √1 − r2]/r)2 + 2), which r = 0.25, β = 0.95, and α = 0.05 then, with 95% confidence and 80% power [17]. Migraine was clinically diagnosed by a neurologist. Participants were included in the study based on the following inclusion and exclusion criteria. Entry criteria: Body Mass Index (BMI) 18.5–30 kg/m^2^, visiting the headache clinic for the first time, and the diagnosis of migraine by a neurologist based on the International Classification of Headache Disorders 3 (ICHD3) criteria. By using a general information questionnaire in face-to-face interviews or according to subject’s medical records we exclude subjects with the history of Cardiovascular Disease (CVD), cancer, diabetes, liver or kidney disease, and other neurological disorders because of possible disease-related changes in diet, along with subjects taking medications that affect serum lipoprotein concentrations (e.g. lovastatin, atorvastatin) or those consuming less than 800 kcal/day (3347 kJ/day) or more than 4200 kcal/day (17,573 kJ/day) (28). As we were unable to assess the dietary intakes of the participants over the past years, we excluded the participants who had changed their dietary patterns during the year before the study start (such as following certain dietary patterns). All participants signed written informed consent forms prior to participation. The study protocol was approved by the local ethics committee: IR. TUMS.VCR.REC.1395.1597.

### Anthropometric measurements

Anthropometric measurements were taken from all study participants. Weight was measured while subjects were minimally clothed (i.e., no jacket, jacket or belt and shoes) using a digital scale, to an accuracy of 100 g. Height was measured using a stadiometer while the participant was standing, without shoes, with heels touching the wall, feet together and head facing forward. BMI was calculated from height and weight data using the equation “weight (kg)/height ^2^ (square meter)”. Waist Circumference (WC) was measured halfway between the lower part of the xiphoid process and the umbilicus, and Hip Circumference (HC) was taken at the greatest anterior projection. Waist and hip circumferences were measured using a flexible plastic tape with an accuracy of 0.5 cm. The ratio between Waist and Hip Ratio (WHR) was also calculated.

### Assessment of dietary intake

Dietary intake over the past year was assessed using a semi-quantitative food frequency questionnaire (FFQ). This questionnaire includes a list of 147 food items, along with the standard serving size for each nutrient [18]. The reliability and validity of this questionnaire has been confirmed in Iran [19].

Foods commonly consumed at home were reported and converted to grams of food per day using the IV nutrition software [20]. Nutrient intake was calculated using Nutritionist IV software modified for Iranian foods based on the United States Food Composition Table of the Department of Agriculture (USDA).

At baseline, scores of intensity (from 0 to 10 on a Visual Analogue Scale (VAS)), and duration were recorded after each migraine attack, regardless of the time of day. Instructions for completing this form were provided by a neurologist. If the participants had a problem completing their diaries, a staff member was available to assist. At the time of recruitment, participants were asked to complete their headache diaries over the preceding month [21].

Physical activity levels (PAL) were measured using the International Physical Activity Questionnaire (IPAQ) and expressed as metabolic equivalent hours/week. Participants were requested to answer questions about the type of physical activity and its intensity as well as the duration of that activity during the day. The sum of the durations of activities in the physical activity questionnaire had to be 24 h. The questionnaire was validated previously [22].

### Calculation of AHEI score

AHEI includes 11 components: fruits, vegetables, whole grains, nuts and legumes, n-3 chain fatty acids, polyunsaturated fatty acids, fruit juice and sugar-sweetened beverages, red and processed meat, trans fatty acids, sodium, and alcohol. Alcohol consumption was not assessed in this study due to lack of data. Scores for consumption of fruits, vegetables, whole grains, nuts and legumes, long-chain n-3 fatty acids and polyunsaturated fatty acids were considered as ‘10’ and ‘0’, respectively, for the highest and lowest amount.

For fruit juice and drinks sweetened with sugar, red and processed meat, trans fatty acids and sodium, respectively, the AHEI score for each component ranged from 0 to 10 for nonadherence to full adherence, respectively, and then participants were categorized into two groups based on the median of their AHEI scores ( median:47, maximum:70 and minimum:23) [15].

### Migraine diagnosis

Based on ICHD3 criteria, episodic migraine was diagnosed by a neurologist. Episodic migraine diagnosis criteria include two types of migraine, with aura and without aura. A diagnosis of migraine without aura, according to the International Headache Society (IHS), can include five or more attacks lasting 4 to 72 h. The headache must have two or more of the following features: unilateral, throbbing, moderate or severe pain, worsening or avoidance of usual Physical Activity (PA), as well as one or more of the following: nausea and/or vomiting, sensitivity to light (photophobia) and sound (ova phobia), feature of migraine with normal aura (with or without headache), Migraine with Brainstem Aura (MBA), hemiplegic, and retinal involvement. MBA is a type of migraine headache with aura, which is accompanied by pain in the back of the head. An aura is a group of warning signs known as a bad headache coming on. Auras may include symptoms such as vertigo, slurred speech, ataxia, tinnitus, visual changes, and/or loss of balance, as diagnosed by a neurologist [23].

### MIDAS and VAS handbook

Migraine Disability Assessments (MIDAS) questionnaire was used to evaluate migraine disability and migraine severity [24]. This questionnaire consists of five questions that evaluate the severity of headache in the last three months. The first to fifth questions of the questionnaire evaluate the decrease in performance caused by migraine. Based on the total score of these five questions, participants are placed into one of four groups: 0–5 (MIDAS grade I, little or no disability), 6–10 (MIDAS grade II, mild disability), 11–20 (MIDAS grade III, moderate disability), 21+ (MIDAS grade IV, severe disability). This questionnaire was previously validated in Iranian populations [25].

Also, visual Analog Scale (VAS) was used to measure pain. The VAS consists of a 10 cm long horizontal line fixed by word descriptors at each end. The patient marks a point on the line that they feel represents an understanding of their current situation. By using a ruler, the score is determined by measuring the distance (mm) on the 10-cm line between the “no pain” anchor and the patient’s mark. A higher score indicates greater pain intensity. Cut points include: mild pain (1–3), moderate pain (4–7), and severe pain (8–10) [18]. This questionnaire was previously validated [26].

### Evaluation of other variables

Age, marital status, education, and job were collected by the researchers through a demographic questionnaire. Physical activity levels (PAL) were measured using the International Physical Activity Questionnaire (IPAQ) and evaluated according to the time spent on light, moderate, high and very intense activities. PAL was expressed as metabolic equivalent hours per week (METs hours per week).

### Statistical analysis

Chi-square test were used to examine the association between AHEI and qualitative variables. Also, independent T-Tests were used to investigate the association between AHEI and quantitative variables (such as dietary intake, age, weight, body mass index, waist circumference, etc.). Mild pain in VAS variable, no disability in MIDAS variable, low adherence of AHEI variable were considered as reference group. Multinomial logistic regression was used to investigate the association between AHEI and Midas and VAS. Also, we used binary logistic regression for association between AHEI with headache frequency. P < 0.05 was considered statistically significant, and SPSS version 24 (SPSS Inc., Chicago, IL, USA) was used for statistical analysis.

## Results

### Participant characteristics

The mean (± SD) age, height, weight, BMI, and physical activity of participants were 34.29 (7.95) years, 1.61 (0.05) m, 67.71 (13.04) kg, 25.88 (4.74) kg/m^2^, 380.03 (599.63) MET/min/week, respectively. The mean ± sd, range, median, min and max of AHEI score were as follow: 45.33 ± 10.52, 47,47,23,70. Except for duration (less than and more than 10 h of migraine), there was no significant association between high adherence AHDI compared with low adherence. However, people with higher adherence to the AHEI compared to lower adherence had better scores for VAS, disability, and headache frequency.

### Participant characteristics among intake of AHEI

The mean intake of AHEI was significantly different among those with higher adherence to AHEI, Table 1. Individuals with higher AHEI adherence had higher energy, total fat, cholesterol, saturated fatty acid, trans, Mono Unsaturated Fatty Acid (MUFA), Poly Unsaturated Fatty Acid (PUFA), oleic acid, linoleic acid, linolenic acid, Eicosapentaenoic Acid (EPA), Docosahexaenoic Acid (DHA), total fiber, riboflavin (B2), pyridoxine (B6), cobalamin (B12), vitamins K, E, D, C, biotin, retinol, lutein, lycopene, sodium, potassium, calcium, iron, phosphor, magnesium, and copper (p-value < 0.001) intakes compared to individuals with lower adherence.

### The association between AHEI and severity, duration, and frequency of migraine headache

Multivariable-adjusted OR and 95% CI for AHEI and migraine VAS, MIDAS, frequency, and duration migraine are shown in Table 2. A significant positive association was found between adherence to the AHEI and headache duration. People with high adherence of AHEI, compared with low adherence, had 43% reduction in headache duration (OR = 0.57; 95% CI 0.34, 0.97; P = 0.03) in the crude model; this association remained significant in the adjusted model (OR = 0.56; 95% CI 0.31, 0.99; P = 0.04). No association was found between the frequency of migraine and AHEI in both crude and adjusted models (OR = 1.19; 95% CI 0.66, 2.14; P = 0.55). No significant association was found between high adherence of AHEI and odds of severe (OR = 0.72; 95% CI 0.31, 1.67; P = 0.45) and moderate headaches (OR = 0.69; 95% CI 0.31, 1.51; P = 0.36). No significant association was found between high adherence of AHEI and odds of moderate (OR = 0.76; 95% CI 0.28, 2.07; P = 0.60) and severe (OR = 1.28; 95% CI 0.53, 3.12; P = 0.57) disability during migraine headache.

**Table 1:** Characteristics of study population based on adherence of AHEI.

Characteristics	Low adherence of AHEI	High adherence of AHEI	
Quantitative variables	Mean ± SD	Mean ± SD	P-value
Age (year)	34.29 ± 7.95	34.34 ± 7.77	0.95
Height (m)	1.61 ± 0.05	1.62 ± 0.05	0.56
Weight (kg)	67.71 ± 13.04	71.44 ± 12.89	0.02
BMI (kg/m^2^)	25.88 ± 4.74	27.23 ± 5.01	0.02
HC (cm)	103.94 ± 11.39	103.91 ± 13.14	0.98
WC (cm)	85.55 ± 12.44	87.45 ± 13.02	0.22
WHR (cm)	0.82 ± 0.06	0.94 ± 0.86	0.13
Physical activity (MET/h/week)	380.03 ± 599.63	437.18 ± 406.03	0.37
Qualitative variables	N (%)	N (%)	P-value
Marital situation			
Single	44 (59.5)	30 (40.5)	0.55
Married	98 (52.1)	90 (47.9)	
Divorced	2 (50)	2 (50)	
Education			
Undergraduate	66 (51.57)	60 (48.42)	0.31
Bachelor	37 (46.8)	42 (53.2)	
Master or higher	40 (56.16)	20(43.83)	
Job			
Employed	50 (50)	47 (66.36)	0.81
Unemployed	95 (60.13)	75 (39.86)	
Migraine severity			
VAS			
Mild	25 (58.1)	18 (41.9)	0.84
Moderate	61 (53.5)	53 (46.5)	
Severe	58 (53.2)	51 (46.8)	
MIDAS			
Without or low disability	19 (54.3)	16 (45.7)	0.35
Mild disability	42 (63.6)	24 (36.4)	
Moderate disability	23 (50)	23 (50)	
Severe disability	60 (50.8)	58 (49.2)	
Headache duration			
≤ 10 h	89 (49.7)	90 (50.3)	0.03
> 10 h	55 (63.2)	32 (36.8)	
Headache frequency			
> 15 days/month	95 (57.6)	70 (42.4)	0.15
≥ 15 days month	49 (48.5)	52 (51.5)	

BMI: body mass index, WC: waist circumference, HC: hip circumference, WHR: weight to hip ratio, VAS: visual analog scale, MIDAS: migraine disability assessment, AHEI: alternate healthy eating index.

Quantitative variables calculated by T-test and presented as mean ± SD.

Qualitative variables calculated by chi-square test and presented as N (%).

P-value < 0.05 was considered significant.

**Table 2:** Dietary intake based on adherence of AHEI

Variables	Low adherence of AHEI (n = 144)	High adherence of AHEI (n = 122)	P-value
	Mean ± SD	Mean ± SD	
**Macronutrients**			
Energy(kcal/d)	2363.62 ± 490.91	2074.48 ± 510.97	< 0.001
Carbohydrates(gr/ day)	296.38 ± 63.57	288.04 ± 73.55	0.32
Protein(gr/day)	80.63 ± 13.24	77.72 ± 18.62	0.15
Total fat(gr/day)	100.57 ± 31.12	75.75 ± 24.77	< 0.001
CHOL(gr/day)	286.45 ± 88.94	198.58 ± 75.26	< 0.001
Saturated fat(gr/day)	34.02 ± 12.51	20.64 ± 7.26	< 0.001
Trans fat(gr/day)	0.0004 ± 0.0009	0.0000 ± 0.0001	< 0.001
MUFA(gr/day)	33.13 ± 11.50	24.74 ± 10.98	< 0.001
PUFA(gr/day)	18.30 ± 8.11	14.99 ± 7.95	0.001
DHA(gr/day))	0.07 ± 0.06	0.10 ± 0.13	0.007
EPA(gr/day	0.01 ± 0.02	0.03 ± 0.04	< 0.001
Oleic(gr/day)	29.78 ± 10.99	22.12 ± 10.81	< 0.001
Linoleic(gr/day)	15.54 ± 7.65	12.62 ± 7.53	0.002
Linolenic(gr/day)	1.38 ± 0.61	0.97 ± 0.55	< 0.001
Total fiber(gr/day)	34.36 ± 12.78	44.58 ± 15.05	< 0.001
Micronutrients			
Thiamin(mg/day)	1.67 ± 0.43	1.73 ± 0.60	0.30
Riboflavin(mg/day)	1.82 ± 0.39	2.02 ± 0.55	0.001
Niacin(mg/day)	22.71 ± 5.11	22.62 ± 6.47	0.90
Pantothenic acid(mg/day)	4.65 ± 0.95	4.87 ± 1.26	0.12
Pyridoxine(mg/day)	1.76 ± 0.37	2.05 ± 0.51	< 0.001
Folic acid(mg/day)	495.31 ± 115.27	568.05 ± 135.23	< 0.001
Cobalamin (mg/day)	4.23 ± 1.34	2.79 ± 1.04	< 0.001
Vitamin K(mg/day)	325.94 ± 364.86	606.29 ± 451.55	< 0.001
Vitamin D(µ/day)	2.36 ± 1.08	1.62 ± 0.86	< 0.001
Vitamin E(mg/day)	11.76 ± 4.97	11.52 ± 4.61	0.69
Vitamin C(mg/day)	105.23 ± 69.50	177.40 ± 90.47	< 0.001
Biotin(mg/day)	23.77 ± 6.77	29.84 ± 10.53	< 0.001
Retinol(mg/day)	632.56 ± 233.56	807.83 ± 355.69	< 0.001
Lutein (mg/day)	1964.91 ± 1632.38	3540.38 ± 2165.51	< 0.001
lycopene (mg/day)	2150.40 ± 1724.83	3624.58 ± 2090.08	< 0.001
Sodium(mg/day)	3859.95 ± 862.72	3610.37 ± 680.13	0.009
Potassium(mg/day)	3548.23 ± 1034.38	4640.25 ± 1507.01	< 0.001
Calcium(mg/day)	1120.75 ± 402.63	1440.21 ± 495.18	< 0.001
Iron(mg/day)	33.36 ± 24.72	51.18 ± 31.21	< 0.001
Phosphor(mg/day)	1359.69 ± 260.59	1433.04 ± 356.01	0.06
Magnesium(mg/day)	363.55 ± 78.54	441.79 ± 137.65	< 0.001
Chromium (mg/day)	0.08 ± 0.06	0.12 ± 0.11	0.002
Copper (mg/day)	1.40 ± 0.28	1.56 ± 0.45	0.001
Glucose	16.41 ± 8.07	15.95 ± 4.53	0.55
Galactose	1.96 ± 0.97	2.14 ± 1.09	0.16
Fructose	18.77 ± 9.28	18.41 ± 5.40	0.69
Sucrose	21.14 ± 8.68	19.53 ± 9.67	0.15

CHOL: cholesterol, MUFA: monounsaturated fatty acid, PUFA: polyunsaturated fatty acid, EPA: eicosapentaenoic acid, DHA: docosahexaenoic acid, AHEI: alternate healthy eating index.

P-value obtained from T-test.

All data are presented as mean ± SD.

P-value < 0.05 was considered significant.

**Table 3:** The association between adherence of AHEI and severity, duration, frequency, and MIDAS of migraine

Variables	Number of participants	Crude		Adjusted	
		OR (95% CI)	P-value	OR (95% CI)	P-value
Headache duration					
≤ 10 h	179	Reference group			
> 10 h	87	0.57 (0.34,0.97)	0.039	0.56 (0.31,0.99)	0.049
Headache frequency					
< 15 days/month	165	Reference group			
≥ 15 days month	101	1.44 (0.87,2.36)	0.151	1.19 (0.66,2.14)	0.555
VAS					
Mild	43	Reference group			
Moderate	114	0.82 (0.40,1.64)	0.603	0.69 (0.31,1.51)	0.360
Severe	109	0.81 (0.40,1.67)	0.583	0.72 (0.31,1.67)	0.450
MIDAS					
Without disability	35	Reference group			
Mild disability	67	1.47 (0.64,3.39)	0.362	1.28 (0.53,3.12)	0.578
Moderate disability	46	0.84 (0.34,2.03)	0.702	0.76 (0.28,2.07)	0.600
Severe disability	118	0.87 (0.40,1.85)	0.721	0.76 (0.31,1.88)	0.561

VAS visual analog scale, MIDAS migraine disability assessment, AHEI: alternate healthy eating index.

All data are presented as OR and 95% Cl by multinomial logistic regression.

P-value for adjustment model: based on energy, age, marital situation, education, job, drinking water.

## Discussion

To our knowledge, this cross-sectional study is the first to explain the association between AHEI and severity, duration, and frequency of migraine headache among women. Adherence to AHEI has a significant association on lower duration of headache even after adjusting for confounding factors, although other variables of severity migraine, such as VAS, MIDAS, and frequency of headache showed a decreasing trend associated with AHEI, these results were not significant.

Our study had similar results to previous studies, such that the frequency of headache appeared to be decreased by diet rich in vegetables, fruits, and potassium, and low in fat [27]. Vascular headache in adults can be affected by many dietary factors, including chocolate, cheese, fasting, alcoholic drinks, and coffee [28–31]. Some studies have shown that occurrence of headaches is related to increased intake of monosodium glutamate [32–34]. On the other hand, a recent study indicated inconsistent evidence on the association between sodium glutamate consumption and headaches [35], and another study showed that monosodium glutamate is responsible for migraine headache in only 2.5% of study participants [31]. In a recent study, women with migraine, compared to women without migraine, had a lower intake of orange vegetables, green vegetables, fruits, whole grains, legumes, and oil [36]. Another study concluded that vegetables, such as broccoli, cabbage, parsley, beets, carrots, and spinach, have active components with an antagonistic ability against CGRP, which can be used to counter migraines, and potentially more effective than commonly administered drugs [37].

Further, in women with migraine, consumption of pro-inflammatory omega-6 fatty acids, as compared to anti-inflammatory omega-3 fatty acids, is associated with an 11-fold increase in the development of inflammation [38]. AHEI includes healthy foods such as fruits, vegetables, whole grains, nuts and legumes, n-3 chain fatty acids, polyunsaturated fatty acids, and fruit juice [15]. The results of meta-analysis revealed that adherence to the AHEI was associated with a reduced risk of hip fracture, depression risk, the risk of all-cause mortality, cardiovascular disease, cancer, and type 2 diabetes mellitus [39–42]. Several clinical studies evaluated the association between serum inflammatory markers and high-fiber diets [43–45] and found that there is an inverse relation between plasma CRP levels and high-fiber diets [46–49]. The mechanisms of these changes are still unknown, however, probable mechanisms include the absorption of glucose, fiber-rich meal modulation of cytokine responses blunting oxidative stress and inflammation, and production of anti-inflammatory cytokines, which can be decreased by high-fiber diets by gut flora [44, 45]. Some studies have indicated that, in migraine patients, the levels of riboflavin, magnesium, and coenzyme Q10s in the brain and plasma were low [50–52]. The deficiency of these micro-nutrients can have a key role in the development of migraine. Riboflavin, magnesium, and coenzyme Q10 play crucial role in the production of energy in the mitochondria [53] and their deficiency can cause mitochondrial dysfunction and then migraine [54, 55]. Furthermore, hypomagnesemia appears to play a key role in the pathogenesis of migraine, whilst magnesium, as a co-factor, is necessary in the generation of ATP.

An inverse association between CRP levels and serum magnesium concentrations has been reported by several studies in children [56], women [57], and obese patients [58]. Also, it has been shown that in participants with total daily magnesium consumption below the RDA, elevated CRP levels were evident [59]. In addition, a cross-sectional study also found an inverse association between total daily magnesium intake and serum CRP levels [60]. However, another recent cross-sectional study found no association between dietary magnesium intake and CRP levels [61]. AHEI, however, is a diet rich in magnesium and riboflavin because of high amounts of vegetables, and it may be efficacious in improving the frequency, or even prevention, of migraine headaches.

To our knowledge, this is the first study to evaluate the association between adherence to the AHEI diet with severity, duration, and frequency of migraine headache among women. In this study, a validated FFQ was used to determine AHEI dietary adherence and assess regular dietary intake. However, there are several limitations that need to be considered when interpreting our findings. The main limitation of the present study was its relatively small number of subjects, as well as the study population being limited to women. Therefore, the results may not be extended to men, whilst the cross-sectional nature of the study precludes causal inferences being made, and thus, we advocate for the conduct of future randomized clinical trials. Additionally, the rationale for the recruitment of women only is that migraine’s prevalence in women (More than 20%) is markedly more than men (more than 10%). One of the inevitable limitations of this type of study is that questionnaire responses are subjectively based on participants’ memory and their perception of pain. Additionally, dietary intake was assessed by a food-frequency questionnaire (FFQ), which is self-reported by the patient and therefore dependent on their memory and also taking food supplements was not asked during this study.

## Conclusion

The present study showed that, with regard to the association between migraine headache and AHEI, people with high adherence of AHEI had a reduction in duration of migraine. However, owing to the study limitations, further clinical trials are suggested to better evaluate and understand this association.

## Data Availability

The data that support the findings of this study are available from Khadijeh Mirzaei, but restrictions apply to the availability of these data, which were used under license for the current study, and are not publicly available. Data are however available from the authors upon reasonable request and with permission of Khadijeh Mirzaei.

## Abbreviations

**AHEI**: Alternative Healthy Diet Index
**ICHD3**: International Classification of Headache Disorders 3
**CVD**: CardioVascular Disease
**BMI**: Body Mass Index
**WC**: Waist Circumference
**HC**: Hip Circumference
**WHR**: Waist and Hip Ratio
**PAL**: Physical Activity level
**IPAQ**: International Physical Activity Questionnaire
**FFQ**: Food Frequency Questionnaire
**IHS**: International Headache Society
**PA**: Physical Activity
**MBA**: Migraine with Brainstem Aura
**MIDAS**: Migraine Disabiliy Assessment Qestionnaire
**VAS**: Visual Analog Scale
**MUFA**: MonoUnsatturated Fatty Acid
**DHA**: DocosaHexaenoic Acid
**EPA**: EicosaPentaenoic Acid
**DHA**: DocosaHexaenoic Acid
